# Aspirin non-adherence in pregnant women at risk of preeclampsia (ANA): a qualitative study

**DOI:** 10.1080/21642850.2021.1951273

**Published:** 2021-08-06

**Authors:** Raya Vinogradov, Vikki Joanne Smith, Stephen Courtenay Robson, Vera Araujo-Soares

**Affiliations:** a The Newcastle upon Tyne Hospitals NHS Foundation Trust, Newcastle upon Tyne, UK; b Population Health Sciences, Reproductive and Vascular Biology Group, Newcastle University, Newcastle upon Tyne, UK; c Midwifery & Health Department, Northumbria University, Newcastle upon Tyne, UK; d Health Technology and Services Research (HTSR), University of Twente, Enschede, Netherlands

**Keywords:** Adherence, aspirin, pregnancy, pre-eclampsia prevention, Theoretical Domains Framework

## Abstract

**Background:**

Antenatal adherence to aspirin prophylaxis is key to reducing the occurrence of a major pregnancy complication: pre-eclampsia (PE). Up to 75% of pregnant women at increased risk of pre-eclampsia do not take aspirin as prescribed. Little research has been done to understand the psychological determinants of aspirin adherence in pregnancy. This qualitative study aimed to explore barriers and facilitators to aspirin adherence in women at increased risk of PE using version 2 of Theoretical Domains Framework (TDF).

**Methods:**

Fourteen women from the North-East of England who declared various levels of non-adherence to aspirin (0–5 of 7 prescribed tablets/week) were interviewed 4–18 months after delivery, using the TDF as a guide. Semi-structured interviews were digitally recorded and transcribed verbatim. A thematic framework analysis was used.

**Results:**

Women exhibited both intentional and unintentional non-adherence and faced multiple barriers at a personal and environmental level. They struggled to initiate, implement and persist in taking medication as prescribed. Women expressed inadequate knowledge about PE and aspirin; they struggled to identify as ‘medication takers’ and relate to the risk factors for PE as identified by the midwife. Significant barriers within the health-care environment were identified; women had difficulties obtaining medication and perceived conflict amongst health care professionals regarding medication safety.

**Conclusion:**

A combination of inadequate knowledge, lack of identification with the risk factors and beliefs about consequences of taking medication were interlinked with other domains, such as environmental context and resonate with the Necessity-Concerns Framework.

## Introduction

Pre-eclampsia (PE) affects 2–5% of all pregnancies (Duley, 2009) and can lead to devastating outcomes; it is the second leading cause of maternal death (Saving Mothers’ Lives, 2011) with an estimated global death toll of 60,000 women per year (Khan, Wojdyla, Say, Gülmezoglu, & Van Look, 2006). Short-term healthcare costs of caring for a mother and baby affected by PE are double those of uncomplicated pregnancy with an excess cost of €2791 per pregnancy (Fox et al., 2017). Mothers affected by PE are more likely to develop cardiovascular disease later in life (Yinon et al., 2010) while offspring are also at risk of developing long-term morbidity (Vatten et al., 2003).

Globally low-dose aspirin is used to reduce the risk of PE. In the World Health Organisation (WHO) recommendations for the prevention and treatment of pre-eclampsia, daily low-dose aspirin is featured as recommended strategy for prevention of PE, (WHO, 2011) while the American College of Obstetricians and Gynecologists (ACOG) suggests 81 mg of daily aspirin to women at risk of PE (WHO, 2011). In line with the NICE guideline ‘Hypertension in pregnancy: diagnosis and management’ (ACOG Practice Bulletin, 2019); in England, women with one major risk factor (chronic hypertension, previous PE, Type 1/Type 2 diabetes mellitus, autoimmune and chronic kidney disease) or more than two minor risk factors (first pregnancy, family history of PE, body mass index (BMI) of ≥35 kg/m2, multiple pregnancy and age ≥40 years) are considered to be at higher risk of PE and are offered prophylactic aspirin therapy (National Institute for Health and Care Excellence, 2019) (75–150 mg taken at bedtime). This is based on high-quality evidence of the effectiveness of a low-dose aspirin for the prevention of PE when started before the 16th week of pregnancy (Roberge et al., 2017a). The results of a recent international RCT of 150 mg of aspirin vs placebo showed a reduction in the incidence of early onset of PE in the aspirin group without increasing the risk of adverse effects (Rolnik et al., 2017a).

In the group of pregnant women at increased risk of PE, non-adherence to aspirin may play a pivotal role in the variability of response to treatment (Vinogradov et al., 2020). A recent subgroup analysis of the ASPRE trial (a randomised controlled trial (RCT) of 150 mg of aspirin to prevent PE) reported the effectiveness of the treatment ranged from 40% in the cohort with adherence of <90% to 75% in those with adherence >90% (Wright et al., 2017). The non-adherence rate to aspirin prophylaxis in pregnancy was suggested to range between 21.4% and 46.3% (Abheiden et al., 2016), which is equivalent to the medication non-adherence rates in non-pregnant patients with chronic disease. However, a recent study suggests that non-adherence to aspirin during standard clinical care may be as high as 75% amongst women at increased risk of PE (van Montfort et al. 2020). Therefore, improving adherence to aspirin in this cohort is crucial to optimise outcomes.

Previous studies of women’s perception of medicine use in pregnancy suggest that women are more likely to avoid the use of medicine during pregnancy due to safety concerns (Nordeng, Ystrøm, & Einarson, 2010) and this is exacerbated by the presence of conflicting information on the internet and insufficient support from health care providers (Ceulemans, Van Calsteren, Allegaert, & Foulon, 2019). Women are more likely to take medicine if they perceive that their medical condition poses a threat to their fetus (Nyholm, Andersen, Vermehren, & Kaae, 2019). Women may view aspirin therapy differently in pregnancy because of its prophylactic use to prevent the onset of PE, rather than the treatment of symptoms of an established disease. Lin et al explored an extended theory of planned behaviour (with addition of action planning, coping planning and relationships with husband) to explain aspirin adherence in pregnant women (Lin, Broström, Nilsen, & Pakpour, 2018). The study supported the proposed theory; however, it could not demonstrate casual relationships amongst variables and no association between subjective norms and intentions to perform the behaviour was found. Although the theory of planned behaviour has been recognised for its contribution to the development of behavioural sciences, it is heavily criticised for its parsimonious approach, limited validity and utility (Sniehotta, Presseau, & Araújo-Soares, 2014). In a recent qualitative study of aspirin use amongst pregnant women (*n* = 6) only one barrier (pill burden) and one facilitator of aspirin adherence (relationship with health care provider) were reported (Shanmugalingam et al., 2020). Although literature on psychological determinants of non-adherence to aspirin treatment is scarce, personal characteristics such as maternal age, smoking status, level of education, parity, ethnicity, and previous history of PE are known to be associated with non-adherence to medication in pregnancy (Ceulemans et al., 2019; Lupattelli, Spigset, & Nordeng, 2014; Wright et al., 2017).

As non-adherence to aspirin prophylactic therapy in pregnancy, is likely to be complex and multifaceted, and having little evidence from other qualitative research in this area, we decided to adopt a wider theoretical approach and to apply qualitative methodology to gather an in-depth understanding of this phenomenon (non-adherence to aspirin in pregnancy).

To synthesise psychological determinants of non-adherence to aspirin in pregnancy the Theoretical Domains Framework (TDF) can be used. The TDF was developed as an overarching framework that integrates behaviour enactment and change theories under a series of domains. The TDF is based on behaviour change theories and models, with the factors associated with behaviour and behaviour change. This framework has been used to identify modifiable barriers, enablers and enactment to behavioural change, as well as to develop theory-based behaviour change interventions (Francis, O’Connor, & Curran, 2012; Michie et al., 2005). The refined framework, containing 14 domains (subsuming 84 constructs (Cane, O’Connor, & Michie, 2012; Francis et al., 2012), has been widely used for behaviour change research across a range of clinical situations (Birken et al., 2017). However, the TDF has not been used to explore factors affecting adherence of pregnant women to prescribed aspirin. Identification of modifiable components of the behaviour may facilitate the development of more effective interventions to increase adherence to medication in the future (Araújo-Soares, Hankonen, Presseau, Rodrigues, & Sniehotta, 2019).

The aim of ‘Aspirin non-adherence in pregnancy – ANA’ study was to gain an in-depth understanding of the reasons for women’s non-adherence to prophylactic aspirin prescribed during pregnancy, by identifying modifiable barriers and facilitators of medication adherence in women at risk of preeclampsia, using the TDF.

## Methods

A qualitative study using semi-structured interviews based on the TDF was conducted. An interview guide was developed, based on the refined TDF (Cane et al., 2012) and subsequently modified following a review by an independent group of women (see interview guide in supplementary materials, Appendix 1).

## Population

Participants recruited from an ongoing study ‘Wave’ (trial registration number ISRCTN41944844), who consented to be contacted for the purposes of further research and disclosed adherence levels ≤5 aspirin tablets per week (out of 7 prescribed) at the time of the 20-week scan, were given the opportunity to participate. Women who experienced perinatal loss were not approached.

**Figure 1.**
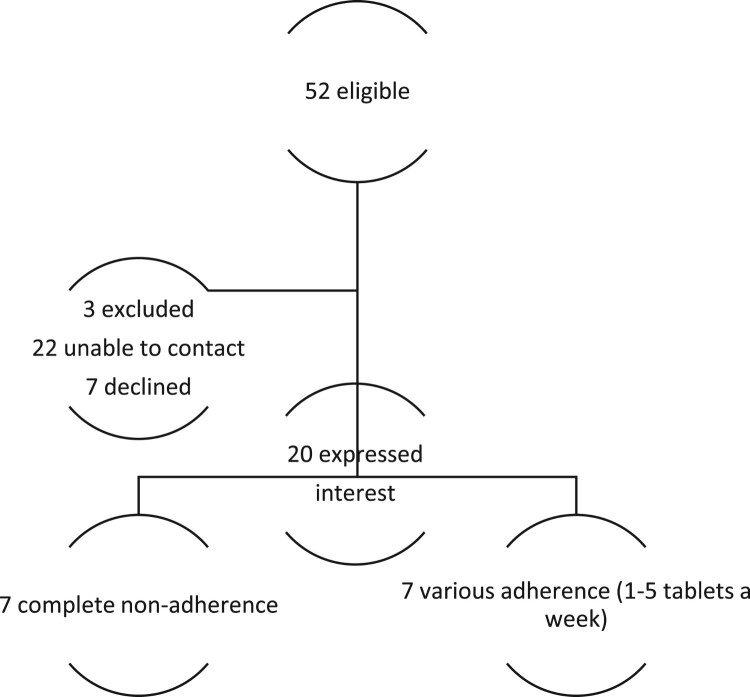
Recruitment diagram.

Out of 52 women meeting non-adherence criteria, 20 women expressed wiliness to participate. We purposively selected women with the lowest levels of adherence to enable us to identify barriers to adherence. We also endeavoured to include participants from different social backgrounds, with diverse risk factors, parental experience and age (see participant’s characteristics in Table 1 and Figure 1 [recruitment diagram]). Although an additional six women expressed an interest in taking part in the study, data collection was stopped following the analysis of 14 interviews, as data saturation was achieved, as agreed at a research meeting. Interviews were conducted 4–18 months following the delivery of the baby (mean 9.4 months). All participants were from North-East of England.

**Table 1.** Participant demographics.

Participant	Adherence level	Ethnicity	Age	Edu	Parity	Child’s age	Risk factor
ANA 1	0/7	WB	31	10	3	14 m	BMI + Fh
ANA 2	0/7	WB	40	13	2	18 m	BMI, CHPT, PIH, SGA, Interval
ANA 5	0/7	Mixed	41	25	0	13 m	BMI, P0
ANA 7	0/7	WB	23	11	1	4 m	PE
ANA 10	0/7	WB	24	9	1	8 m	BMI, PE
ANA 11	0/7	WB	36	13	1	4 m	BMI, Interval
ANA 14	0/7	WB	33	18	1	7 m	PIH?PE
ANA 4	1/7	WB	40	13	0	6 m	P0, Fh
ANA 6	3/7	WB	29	11	1	12 m	PE, Fh
ANA 8	4/7	WB	31	14	2	7 m	T2DM
ANA3	5/7	WB	33	18	1	11 m	PE, SGA
ANA 9	5/7	WB	36	22	1	15 m	PIH, Fh
ANA 12	5/7	WB	24	16	0	8 m	T2DM, P0
ANA 13	5/7	WB	24	15	0	4 m	BMI, P0
Values expressed in mean (min and max)			31.8 (24–41)	14.7 (9–25)	1 (0–3)	9.4 (4–18)	

Notes: Adherence expressed in number of pills taken over 7 days preceding 20 weeks visit.

Ethnicity: WB – with British.

Education expressed as number of years in full time education.

Parity number of livebirths at the time of the index pregnancy.

Child age expressed in month and reflect the time lag since the end of the index pregnancy.

Risk factors: PE – pre-eclampsia in previous pregnancy, BMI – Body Mass Index > 35 kg^2^/cm, Fh – family history of PE, CHPT – Chronic hypertension, PIH – pregnancy induces hypertension, SGA – small for gestational age infant, Interval – Pregnancy interval > 10 years, P0 – no previous livebirths over 24 weeks gestation, PIH – pregnancy induces hypertension in previous pregnancy, T2DM – Type 2 Diabetes Mellitus.

## Ethical considerations

The study was reviewed and given a favourable opinion (NHS REC ref 19/NI/0139). All women gave informed written consent to participate in the study.

## Researcher’s characteristics

Interviews were conducted by a trained researcher (RV). RV (BA, M Clin Res) is a female senior research sonographer and was the lead researcher for both the ‘Wave’ and ‘ANA’ studies and therefore had an opportunity to meet participants and establish relationships as a researcher. There was at least a 9-month interval between the Wave and ANA study visits. All participants were aware of RV’s research role. The rest of the research team had no contact with the participants and had only access to the anonymised transcripts for the purposes of data analysis and to assure quality.

## Data collection

Women were offered an option to be interviewed in person, by phone or using Skype. Duration of the interviews varied from 1 to 2 h. Women were asked to provide a narrative regarding their experiences of aspirin use (or non-use) and were then guided by the interviewing researcher to discuss specific aspects of their experiences (as per the interview guide) if not previously mentioned by participants. The duration of individual interviews was determined by the quantity of information women were willing to share. Length of each interview was also influenced by participant personal characteristics such as communicativeness and by the relevance of questions to women’s experience i.e. in cases where women have experienced intentional non-adherence (decided not to take aspirin) questions regarding their experience with aspirin intake were omitted.

No repeat interviews were performed. Interviews were audio-recorded and transcribed verbatim. Field notes were collected and used to assist researchers to immerse in the data. Transcribed interviews underwent participant checks and were anonymised. Data were managed using NVIVO 12.

## Analysis

As we aimed to describe and interpret what is happening in particular settings, i.e. what barriers and facilitators women face in adherence with aspirin, a thematic framework analysis (TFA) was selected to analyse the data. TFA is set to answer a specific question (to understand barriers and facilitators of adherence), it is appropriate to be conducted within a limited timeframe, uses a pre-designed sample and addresses a priori issues (Araújo-Soares et al., 2019). TFA was conducted using five analytical stages: familiarisation, identifying a thematic framework, indexing, charting, and mapping and interpretation (Araújo-Soares et al., 2019; Srivastava, 2009). As both the interview guide and the framework were constructed using the TDF, care was taken to perform inductive data coding first to ensure all themes were identified. Once coded, data were sorted into the thematic framework (see final coding frame in Appendix 2). Analysis of the interviews was conducted by xx and xx in parallel with data collection. During this period, research clinics were held regularly amongst the members of the research team (RV, VS, VAS, SV) with the aim to review coding, the framework, to resolve disagreements, and to identify data saturation as it emerged.

## Results

The results presented in this section under each of the TDF domains can be examples of either barriers or facilitators of adherence. A selection of representative quotes is provided to illustrate the domains and relevant constructs within the domain (see Appendix 3, Table 2 and Table 3 for a wider selection of quotes representing barriers and facilitators to adherence in this cohort).

## Types of non-adherence

Women exhibited both intentional and unintentional non-adherence.

In the case of intentional non-adherence some women made an explicit decision not to take aspirin as prescribed:

If I was given a prescription, I must have put it straight in the bin. ANA 14 (33 yo, P1, prev PE, 0/7)

Others implied that they had no intention to take it:

I haven’t even got the prescription yet. I kept saying I’ll get it tomorrow. I didn’t think I need it, so I didn’t take it. ANA 1 (31 yo, P3, BMI + Fh, 0/7)

Some women made a decision to take aspirin, but struggled to keep up with the regular medication schedule i.e. exhibited unintentional non-adherence:

Sometimes a day would go past, and I would realise I hadn’t taken it. And it wasn’t really so much because I didn’t want to or because I didn’t understand why. ANA 3 (33yo. P0, PE, SGA, 4/7)

Intentions dictated the type of non-adherence exhibited by women (intentional vs unintentional). However, participants’ intentions were subject to change across time, making the distinction between intentional and unintentional adherence difficult; some women did not make an attempt to review their initial intention about whether to take aspirin, whilst others changed their intentions in response to environmental and/or physiological events, further independent learning or interactions with health care providers.

## Determinants of non-adherence as defined by medication taking processes

Women’s narratives fed into three main themes aligned to medication taking processes (Vrijens et al., 2012) (see Table 4 in Appendix for summary of the themes):

1. Decision-making (necessity – concerns balance and restricted timeline of pregnancy)
2. Implementation
3. Discontinuation

### Decision-making (necessity-concerns balance)

Decisions regarding aspirin were based on a very fine balance of beliefs held by women about the necessity of the medication and their concerns regarding the use of aspirin during pregnancy. Domains such as Knowledge (poor knowledge about PE), Identity (lack of identity with at risk group), Social Influence (not a social norm), Optimism (unrealistic optimism), and Reinforcement (lack of active reinforcement) contributed to a sense of low necessity to take aspirin. Concerns were fortified by domains such as Knowledge (poor knowledge related to medication), Beliefs about consequences (concerns about effects of aspirin on both the mother and a baby), Environmental context (perceived interprofessional disagreement).

## Necessity

Participants demonstrated limited knowledge about pre-eclampsia and the potential consequences of developing the condition. They discussed having and wanting to maintain an optimistic outlook about the progression of their pregnancy and held misconceptions about the potential benefits of taking aspirin as a preventative medication, which led to women having a reduced sense of necessity about taking aspirin.

The degree of knowledge about PE varied depending on whether women had been diagnosed with the condition previously (had experiential knowledge), knew someone who had the condition (vicarious knowledge) or had theoretical knowledge (had heard or read about it).

Pre-eclampsia … I’ve heard of it. It is not where the placenta blocks the birth passage or something like that … I’ve heard of it … I never really … no …  ANA 2 (40 y/o, P2, multip risks, 0/7)

It’s a blood thinner isn’t it? I am assuming it keeps your heart pumping at the right level and your blood pressure down. ANA 4 (40 y/o, P0 + Fh, 0/7)

I think I had good feeling that it was just never going to happen. I just didn’t think I would ever have pre-eclampsia. ANA 12 (24 y/o, P0, T2DM, 5/7)

Experiencing a low sense of necessity to take aspirin was exacerbated by a lack of personal identification with the risk factors identified by health care professionals. This manifested in women not recognising the need to take the medication. For some women, an inability to relate to the PE-related risk factors discussed by health professionals led to strong negative reactions to being classified as having a ‘high-risk’ pregnancy as women felt stigmatised by attributingto their labels.

I wouldn’t say it’s got anything to do with your weight personally cos it can happen to anyone like the thinnest of people can have really high blood pressure and have blood clots. So, I think they put the stigma on people that are overweight. Everyone’s different. ANA 11 (36 y/o, P1, BMI+ interval, 0/7)

For some women, this was also influenced by the fact that they regarded themselves as someone who does not easily resort to taking medicines. Even women with underlying, chronic medical conditions seemed unable to identify themselves as ‘medication takers’.

I don’t like taking medicine, but I don’t even like taking paracetamol for headaches and stuff. No, it was fine I can take it I just, I don’t like my body relying on medicine. Like if I’ve got a headache, I don’t like taking paracetamol I’d rather my body gets rid of it on its own. ANA 7 (23 yo, P1, prev PE, 0/7)

I felt like, I felt like a pill bottle: if you shake me, I would rattle. Cause I don’t really take medication. If I have a headache, I don’t like go and take paracetamol straight away. I’d put up with it for a while before I took anything for it. ANA 8 (31 yo, P2, T2 DM, 4/7)

Furthermore, women made a clear distinction between the benefits of taking aspirin and other preventative medicines such as folic acid and vitamin D. Vitamins were viewed by women as an essential support to a growing fetus and commonly used, whereas, aspirin was perceived as an uncommon medication to use during pregnancy with little or doubtful benefit.

When you take your folic acid, you are taking the folic acid for the baby -you are not taking it for you … It’s drummed into you and everything you read …  you are good mum if you take your vitamins sort of thing, whereas the discussion about aspirin is not, well … it didn’t feel like it was about that. It was about keeping the mum healthy and it’s the same with lots of medication. If people don’t feel like there are going to get a benefit from it especially a non-visible benefit from it then what’s the point? ANA 14 (33 yo, P1, prev PE, 0/7)

She (a friend) told me she hadn’t really been taking them, only in hospital. So, she didn’t know the benefits of taking them. ANA 8 (31 yo, P2, T2 DM, 4/7)

Interviews highlighted a perceived lack of professional support and reinforcement of the importance of aspirin adherence at the most crucial times during pregnancy, such as at the start of aspirin prescription or at the time of significant events. Furthermore, some women experienced a lack of coherence between hospital and community antenatal care.

The midwives didn’t want to take any responsibility over that. They are quite happy to push vitamins folic acid. But whereas if you had a midwife saying this is good for your baby and I am going to push this. And your community midwives that you had built a relationship with then you might see it slightly differently and take it differently. But it was very … in my experience very much a, that’s a hospital decision … very separate … Considering there was one person being looked after and I am same person whether I am in the hospital or with a community midwife there were very different agendas, very different agendas. ANA 14 (33 yo, P1, prev PE, 0/7)

In addition to the complexities described above, the decision-making process was complicated by the restricted timeline of pregnancy, in particular the short interval between women discussing the option of aspirin prophylaxis with an obstetrician and needing to make a decision about whether to take aspirin. This was dictated by a need to start aspirin early in pregnancy (by week 16) in order for it to be effective. Women also mentioned there was an ‘information overload’ (or a cognitive overload) during consultations with obstetricians and midwives, preventing women from being able to effectively consider the relevant issues and make an informed decision.

You don’t have time to kind of like go through it all because you don’t know what that’s going to say. So initially you’d say yeah okay then but kind of like go home you sit downright okay what am I doing I’m taking this right what does it say, what is it for, what is, what does it do. ANA 5 (41 yo, P0, BMI, 0/7)

## Concerns

A number of concerns were raised by participants about the effects of aspirin intake related to both maternal and fetal health.

I think one risk I didn’t really get to grips with would be the increased risks of like in terms of haemorrhage and bleeding and stuff if you are on aspirin … But as a lay person, I can image if I take that aspirin does that mean I am going to bleed more when I have my baby. ANA 14 (33 yo, P1, prev PE, 0/7)

I wasn’t wanting to take them in case like she got addicted … . I was concerned about heart defect or something, but I didn’t actually question it. ANA 8 (31 yo, P2, T2 DM, 4/7)

Women revealed experiencing mixed messages from health care professionals regarding aspirin intake during pregnancy and perceived it as a disagreement amongst health care professional regarding the safety of aspirin use in pregnancy. An apparent lack of agreement between health care professionals about the safety of aspirin seemed to increase women’s concerns further and contributed to women exhibiting intentional non-adherence to the prescribed medication.

Do you know there’s obviously some kind of risk as to why, if it was that good for you if you’re high risk why would the pharmacies like not be willing to sell you aspirin when you’re pregnant? So, there’s some kind of, do you know what I mean? ANA 11(36 y/o, P1, BMI+ interval, 0/7)

I just went to the GP’s and told them that I had ran out and asked them if I could have some more on a repeat prescription and they told us I couldn’t because I was pregnant […] No, cos I kind of knew that you weren’t (supposed to take it) cos it’s written on the box. So, I knew you weren’t allowed to take it. ANA 7 (23 yo, P1, prev PE, 0/7)

Further concerns were reinforced by the presence of conflicting online information accessed by women when seeking information independently:

I did read a forum cos I’d Googled aspirin during pregnancy and there was a lot of mixed people. Some people were like oh yeah it’s gonna help your pregnancy you know if you’re high risk then other people were saying well actually I don’t really feel that good and I feel quite uncomfortable taking it when they say you shouldn’t be taking stuff like during your pregnancy. And someone had actually wrote the same thing that I thought why pharmacies are reluctant to give aspirin to pregnant people. ANA 11 (36 y/o, P1, BMI+ interval, 0/7)

### Implementation

Once a fine decisional balance was reached, women who decided to take aspirin were facing further barriers that contributed to non-intentional adherence.

A very prominent environmental barrier, reported in most of the interviews, related to access to medication itself. Having no medication to hand may be one of the key factors in unintentional non-adherence, as no matter how determined women are, if they have no access to medication, they are unable to take it. This occurred because of challenges in obtaining a prescription or collecting medication in a straightforward and timely manner.

Environmental barriers within the hospital context meant that some women left the hospital without medication and were unable to obtain it until their next hospital visit, usually 8 weeks later. Some women continued to face this challenge as they struggled to replenish medication through repeat prescription or by getting it from the pharmacy.

Yeah, I got the prescription on the day I had my 12 weeks scan and I took it to the pharmacy and they said it wouldn’t be available until the next day and at that point I lived in xxx and I didn’t have any way to get there and I never ended up picking it up honestly … until … I can’t remember. I think it was after my 20 week and I started the aspirin a couple of days before my 20-week scan, so that was my reason of not taking it, up until 20 weeks. ANA 13 (24 yo, P0, BMI, 5/7)

(Replenishing medication) it was really difficult. I took it to the doctors, but they didn’t put it on the prescription, so I then I had to ring up my hospital too and get my medication cos the doctors didn’t have it on their system. This happened a couple of times to be honest and I did go without medication for a few days because like it was so much of a hassle to try and get it … . ANA 8 (31 yo, P2, T2 DM, 4/7)

Poor memory and attention seemed to form another major barrier to unintentional non-adherence. Women felt that their ability to focus during pregnancy was affected, referring to this as ‘pregnancy fog’. Women suggested that lack of habit, forgetfulness, physical and cognitive overload were the main reasons for missing their medication, while lack of symptoms of the disease and consequently no sense of symptoms relief contributed to unintentional non-adherence.

It was more to the end of my pregnancy when I started missing them. I was getting too tired and I was just going to bed and forgetting. ANA 6 (29 y/o, P1, prev PE + Fh, 3/7)

I think, perhaps, I am not in very much in the habit of taking things. So sometimes a day would go past and I would realise I hadn’t taken it. It was just that the day had gone pas and in all its business and general kind of chaos, sometimes, with my eldest and I just hadn’t got to the point where I did manage to take it. Perhaps simply because I am not in a habit of taking it [ …] no symptoms would arise if I didn’t take it, kind of thing. Forgetting it didn’t lead to any incident’s kind of … or symptoms I suppose, which would make you to have the medicine. ANA 3 (33yo. P0, PE, SGA, 4/7)

Many women lack regular medication taking experience as they have either not taken regular medicines before or never mastered this skill and had low beliefs about their capabilities to do so.

I am not really a tablet person. ANA 10 (24 yo, P1, Prev PE + BMI, 0/7)

I always found taking medication quite difficult. ANA 13 (24 yo, P0, BMI, 5/7)

I’m no good at taking tablets even the folic acid tablets and stuff I didn’t really take them. Yeah, I think I’m just generally bad at taking tablets [… ] I would give it a go but I would probably be the same …  ANA 7 (23 yo, P1, prev PE, 0/7)

### Discontinuation

Critical incidents were often responsible for women discontinuing aspirin, creating a novel state of intentional non-adherence or reducing the levels of motivation to adhere. Events such as a change in their medical condition, significant social issue such as housing problems and loss of a family member were coded as critical incidents affecting adherence.

But it was I think when I got there (to the hospital) and it was the bleed, it (aspirin) wasn’t the first thing that came to my mind. It was when they started asking me questions about what I take, what I was doing, you know … you kind of like go back and go; right, okay, I was asleep, so there’s nothing there … What do I take? They’re like: aspirin, why do you take aspirin? And I told them. And I was like, right, okay, and then it came to me, you know, it’s a blood thinner duh, duh, duh, duh, duh, duh and then you start. ANA 5 (41 yo, P0, BMI, 0/7)

I lost my sister when I was 8 weeks pregnant and that was a very strange one because none of my family knew, so dealing with that was a bit intense. I think the worst things that could happen, we also had to move out of our flat …  ANA 13 (24 yo, P0, BMI, 5/7)

In instances related to a change in medical condition, some women in this cohort proactively sought additional information related to use of aspirin, and although we found no apparent evidence of reinforcement of adherence to aspirin from health care professionals, there was evidence of unconditional support from relatives, irrespective of womens’ decision about whether to take aspirin or not.

 … the only person I’d spoke to was my partner and he said you know your body and you know what’s right for you and he says so if that’s what you want to do then I support whatever you want. And he said only you can make the choice if you’re not happy taking it then don’t take it. He says there’s plenty of women that go through pregnancies and don’t take it. ANA 11(36 y/o, P1, BMI+ interval, 0/7)

## Facilitators of adherence

TDF domains Skills and Behaviour regulation seemed to facilitate adherence behaviours amongst some of the women with unintentional non-adherence.

Women who had previously taken prenatal vitamins or oral contraceptive pills found the skills associated with taking those transferable and useful in taking aspirin.

So, it wasn’t a major obstacle, do you know what I mean it wasn’t a hindrance to have aspirin cos I was already taking my vitamin D, so it was just an extra thing to take. ANA 11 (36 y/o, P1, BMI, interval, 0/7)

In addition to developing a routine, some women also used reminders, calendars and pill boxes to support adherence and establish new routines/habits, reducing unintentional non-adherence.

He (partner) went out to Asda and brought a medicine box and he used it every Sunday and fill it up so that everything was in for the days and times that I needed it. ANA 12 (24 y/o, P0, T2DM, 5/7)

## Summary of the findings

Women exhibited both intentional and unintentional non-adherence to aspirin use during pregnancy. Barriers to adherence were identified in all the domains conceptualised within the TDF, however the domains of ‘Knowledge’, ‘Social (and professional) role and identity’, ‘Optimism’, ‘Environmental context and resources’, ‘Social influence’, and ‘Beliefs about consequences’ seemed to dominate, indicating that women seem to constantly negotiate necessity beliefs and concerns regarding the use of aspirin within the healthcare system which is inadequately adapted to women’s needs in relation to supporting adherence.

Women often knew surprisingly little about PE or prophylactic therapy with aspirin. At the same time women did not see themselves as ‘medication takers’ and could not identify with the PE-related risks ‘assigned’ to them. It seems that little understanding of the condition, the possibility to prevent it, an inability to identify with the risk factors and an optimistic outlook of the pregnancy led to a feeling of reduced need to engage in preventive behaviours, namely the intake of aspirin. This can be seen by data collected and presented around the domains Knowledge, Social Role and Identity (Identity) and Optimism (unrealistic), leading to a weak sense of necessity.

There were also barriers inherent to the system. Once prescribed aspirin, women faced multiple environmental barriers within the hospital and in the community, precluding them from simply obtaining the medication.

Although, aspirin is prescribed by a consultant obstetrician within hospital setting, women were questioned again, when interacting with other health care professionals (such as pharmacists and general practitioners) and asked whether they really needed to take it. Women perceived those mixed messages as intragroup conflict amongst health care professionals regarding the safety of aspirin use in pregnancy. Those concerns were magnified by a warning printed on aspirin packaging inserts against aspirin use in pregnancy. Women were particularly worried about the consequences of aspirin use to themselves and their unborn babies. Further, women made a clear distinction between folic acid, iron, vitamin D and aspirin by attributing status of essential nutrients needed to ‘help the baby’ to folic acid, and vitamin D and the status of ‘medication’ to aspirin.

This highlights important domains: ‘Environmental context and resources’ in particular related to organisational culture and climate within the NHS, ‘Beliefs about consequences’ build upon limited knowledge and the observations of other health care professionals’ reaction to aspirin prescription requests (‘aspirin might be bad for me and baby as different HCPs give me different messages’), lack of identification with a person that is at risk of pre-eclampsia and unrealistic optimism. All these can therefore lead to lower levels of adherence to aspirin.

Adherence to aspirin seemed to be very sensitive to changing contexts during the pregnancy. Expected and unexpected ‘physiological’ changes change in emotional status and family circumstances seemed to play an important role in both intentional and unintentional non-adherence. At the time of these unexpected changes women reported discontinuing the medication or taking it less regularly. ‘Salient events / critical incidents’ related to the change in women’s behaviour related to aspirin adherence were coded within ‘Environmental context and recourses’. Although ‘Salient events/critical incidents’, such as moving house, losing a close relative or developing a new medical condition, seem to have compromised adherence to aspirin amongst interviewed women, they could have provided an opportunity to intervene if seized by HCPs under Make Every Contact Count initiative (Health Education England Making every contact count, n.d.), however the lack of aspirin adherence reinforcement by those HCPs with closer contact with the women was apparent and noted and interpreted by the women as: ‘this is not that important otherwise I would be asked about this’.

Memory and attention were responsible for the unintentional non-adherence in this cohort. Women stated that they suffered from cognitive overload during pregnancy alongside mental and physical tiredness that in turn affected their ability to concentrate on the task and remember to take the aspirin. This, in turn, prevented them from forming a habit.

## Discussion

There is a plethora of literature reporting aspirin use in pregnancy both in terms of safety and clinical effectiveness for the prevention of PE and other conditions (e.g. small for gestational age infant, stillbirth, and prematurity) (Bujold et al., 2009; Rolnik et al., 2017b; Roberge et al., 2017b). However, the importance of adherence to aspirin has only recently been recognised (Abheiden et al., 2016; Navaratnam, Alfirevic, Pirmohamed, & Alfirevic, 2017; Wright et al., 2017). There are only a few studies that explore factors and/or determinants of non-adherence in pregnancy. In this study, we have identified important barriers to aspirin adherence in pregnant women. Although women’s experience of pregnancy is complex, the factors that influence aspirin adherence seem to fit a conceptual model of Necessity – Concern Framework (Horne et al., 2013) which has shown that patients with strong concerns about the intake of a specific medicine and weak beliefs about the necessity of the medication are less likely to adhere to the prescribed treatment (Phillips, Diefenbach, Kronish, Negron, & Horowitz, 2014). This is reflected in the way woman negotiate their perception of necessity (knowledge, lack of identification with the risk factors, lack of reinforcement) and concerns regarding their own and babies’ health as a result of aspirin use during pregnancy (i.e. environmental barriers, beliefs about consequences).

Another factor that precluded women from adhering to aspirin preventative treatment by both reducing the sense of necessity and increasing concerns, was the lack of easy access to medication. This creates a non-verbal message suggesting potential safety concerns amplified by suboptimal knowledge about aspirin amongst pregnant women.

Knowledge about the medicine and the condition lays a foundation stone for patients’ perceptions of the prescribed medicine both in obstetric and non-obstetric studies (Harrold et al., 2012; Magadza, Radloff, & Srinivas, 2009; van der Wal et al., 2006). Knowledge clearly has an important role and is a core part of another adherence-related model, the model of ‘Informed adherence’ outlined by Michie et al. (Marteau, Dormandy, & Michie, 2001).

Women’s inability to relate to their risk factors and to the role of a ‘medication taker’ has been described in several obstetric studies (Fenn, 2019; Gupton, Heaman, & Cheung, 2001). This inability to identify with someone who needs medication may be more pronounced during pregnancy as pregnancy is often perceived as a natural/healthy state and is less likely to be perceived as a time when medication is required. A recent publication by van Montfort et al. suggests that personalisation of the risks may improve adherence to aspirin in pregnancy (van Montfort et al. 2020). This corresponds with wider work in non-obstetric settings advocating for personalised adherence interventions (Petrie, Perry, Broadbent, & Weinman, 2012).

Non-adherent women exhibited unrealistic optimism about their chance of developing PE, presenting narratives that indicated that in order to enjoy pregnancy they were making a decision not to think about risks; doing this led them to adopt an optimistic approach. Women were using ‘external locus of control’ and ‘denial’ to cope with the stress of pregnancy risks instead of adopting active coping strategies. This approach to coping could result from a sequence of events that women face during pregnancy; ‘booking’ processes where there is a time lag between identification of the risk (stress) and the provision of information regarding preventative strategies (coping), highlighting a need for appropriate and timely support provision. In the UK midwives identify risk factors for PE as early as 8–9 weeks gestation. However, aspirin is not usually provided until weeks later when women meet their obstetrician (typically at 12–14 weeks’ gestation). Therefore, women may be aware that they are at increased risk but unable to progress with effective coping for a significant period of time. This could be potentially overcome by the introduction of preventative strategies at the time of risk identification. A recent study of factors influencing adherence to aspirin in high-risk pregnancies by Shanmugalingam et al highlighted the importance of improved communication to support understanding and allay concerns about potential teratogenic effects of the medication (Shanmugalingam et al., 2020). Aspirin has gained a ‘trustworthy’ status over decades of extensive use in the prevention of hypertension and cardiac disease. The ‘trustworthy’ status of the drug has been shown to facilitate adherence (Eborall & Will, 2011), however, our study suggests that aspirin use during pregnancy is not yet trusted by pregnant women or some health care professionals. Provision of timely education could be useful in the future as has been shown to reduce concerns, improve understanding of the condition and medication and subsequently improve adherence to the medication (Viswanathan et al., 2012).

The preventative nature of aspirin use in this group of women (i.e. it is given to prevent the disease from developing rather than relieve the symptoms of the disease) seems to reduce the perception of the necessity to take aspirin. Eborall et al. suggest that in cases of a drug used to prevent disease, further legitimisation from a health care professional is required (Eborall & Will, 2011). Women in our study received mixed messages from health care providers and perceived an interdisciplinary conflict regarding the use of aspirin in pregnancy. This was compounded by a warning against the use of aspirin in pregnancy on the medication insert, resulting in insufficient legitimisation, despite receiving an aspirin prescription from an obstetrician.

Lin et al. have attempted to explain aspirin non-adherence in pregnancy using an extended Theory of Planned Behaviour and suggested that the woman’s relationship with her husband was important in mediating subjective norms and therefore affected adherence (Lin et al., 2018). In our study women received ‘unconditional’ support from their partners and significant others regarding medication, including the decision to discontinue. Hence, we suggest social support may act as both a facilitator and a barrier to adherence. Some women expressed clear independence in their decisions. Although the relation between social support and adherence is not unidirectional in intentional non-adherence, women that exhibited unintentional non-adherence found partners instrumental in supporting routines and reminding women to take their medication. This seems to indicate that engaging a partner around the need to take prophylactic aspirin would be relevant in supporting adherence.

The initial decision-making process is critical for adherence. In the case of PE there is also the issue of a need to begin prophylaxis early in pregnancy (12 weeks) meaning that an inability to make a timely decision can seriously compromise the effectiveness of the aspirin treatment. We were able to demonstrate that in this complex process women weigh their concerns regarding their baby’s and their own health against perceived necessity of the medication while having little knowledge and professional support. Supporting women with decision-making prior to treatment initiation, using principles of shared decision-making, is more likely to lead to better engagement with therapy (Elwyn et al., 2010).

Adequate adherence is often associated with the use of strategies to overcome memory and attention issues. Establishing routines and habits as well as introducing cues for action can overcome unintentional non-adherence (Gardner, Lally, & Wardle, 2012; Lally, van Jaarsveld, Potts, & Wardle, 2010; Michie et al., 2013). Stability of routines as well as beliefs about medicines alongside good professional support has been highlighted by Chambers et al. in a qualitative research of adherence in stroke survivors (Chambers et al., 2011). Regular health care professional support and attention to medication adherence is required in order to support women throughout the implementation of the correct drug regimen, especially during any critical events, such as hospital admissions, changes in woman’s physical environment or emotional status. Reinforcement is crucial in order to establish and maintain a new behaviour. It is important to increase the opportunities to support women that persevere with the treatment as well as to provide women with appropriate support.

## Strengths and limitations

We were able to access a hard-to-reach population of non-adherent women not previously researched. We approached the study without a specific theoretical pre-conception, instead, we used the TDF as an overarching framework and have arrived at a set of constructs that are likely to explain aspirin adherence in pregnancy using an inductive analytical process.

The obvious limitation of the study is that we only interviewed women who exhibited non-adherence behaviours in pregnancy. Therefore, the study results reflect more barriers and less facilitators of adherence. All participants were from a single tertiary hospital in the North-East of England. It is possible that some of the barriers related to environmental context and resources are characteristic of this part of the country and more needs to be done to understand this.

It is important to highlight that women were interviewed at least 4 months after delivery of the baby and therefore there is the possibility of recall bias. The decision to interview women postnatally was made in order to reduce unnecessary worry regarding the effect of women’s non-adherence on their pregnancy outcome.

## Conclusion

The study has shown that theoretical underpinning of Necessity-Concerns Framework plays an important role in intentional non-adherent behaviours amongst pregnant women at risk of PE while deficiency in memory and attention was prevalent in unintentional non-adherence.

We have identified important issues that present major barriers to adherence:

- Access to medication
- Perceived necessity of medication
- Beliefs about consequences of taking medication linked to the health professional support and advise

## Implications for clinical practice

- Clear pathways should be in place for women to obtain medication.
- It is necessary to improve knowledge, skills and to familiarise women with appropriate behaviour regulations in order to improve adherence.
- Regular medication adherence support should be available for women throughout pregnancy at the time of initiation of the treatment to support the decision-making process and formation of habitual behaviour.
- Partners and other significant members of the women’s support system should be involved in conversations about aspirin use in pregnancy.
- Adherence should be carefully reviewed at the time of any medical or social change.

## Research recommendations

Further research is needed to investigate views of health care professionals around the use of aspirin in pregnancy and perceived level of support provided to women. There is a scope to conduct a wider investigation of determinants of non-adherence to include participants from other regions in the country. Behaviour intervention needs to be developed, evaluated and implemented to support adherence to aspirin amongst women at risk of PE.

## Supplementary Material

Supplemental Material
