# Supplementary material for: Aspirin non-adherence in pregnant women at risk of preeclampsia (ANA): a qualitative study
Source: Health Psychol Behav Med. 2021 Aug 6;9(1):681–700. doi: 10.1080/21642850.2021.1951273 (PMC8354178; doi:10.1080/21642850.2021.1951273)
Supplement: Supplemental Material [file RHPB_A_1951273_SM7448.zip › Appendix 2 ANA Full coding frame.docx]

# Coding frame

| TDF domains | Emerged themes | | |
| --- | --- | --- | --- |
| 1. Knowledge | Knowledge about the disease | Experiential |  |
|  |  | Vicarious |  |
|  |  | Theoretical |  |
|  |  | No knowledge |  |
|  | Knowledge about risk factors |  |  |
|  | Knowledge about aspirin | Task environment |  |
|  |  | Necessity |  |
|  |  | Procedural knowledge |  |
|  | Informational needs* | Informational needs amongst pregnant women* |  |
|  |  | Informational needs amongst partners * |  |
|  |  | Nature of informational seeking behaviour* |  |
|  |  | Sources of information used* |  |
|  |  | Desirable format of information* |  |
| 1. Skills | Coping strategies | Passive vs active |  |
|  | Experience | Gives medication to others |  |
|  |  | Vicarious |  |
|  |  | Already takes medication regularly | Chronic illness |
|  |  |  | Lo vitamins |
|  | No skills |  |  |
| 1. Social/Professional role and Identity | Identity related to medication taking |  |  |
|  | Identifies with the risk factors | Able to relate |  |
|  |  | Unable to relate |  |
|  | Social identity | Towards unborn |  |
|  |  | Towards other children |  |
|  | Professional identity |  |  |
| 1. Beliefs about capabilities | Self esteem |  |  |
|  | Beliefs related to medication |  |  |
|  | Perceived behavioural control |  |  |
| 1. Optimism | Chances for getting the disease | Unrealistic optimism vs pessimism |  |
|  | Medication works | Optimism vs pessimism |  |
| 1. Beliefs about consequences | Of taking medication | For mother | Benefit vs regret |
|  |  | For baby | Benefit vs regret |
|  | Of not taking medication | For mother | Benefit vs regret |
|  |  | For baby | Benefit vs regret |
| 1. Reinforcement | Punishment/sanctions |  |  |
|  | Reverse reinforcement | Reinforcement of non-adherence |  |
| 1. Intentions | Intentions |  |  |
|  | Stability of intentions |  |  |
| 1. Goals | Goals | Distal |  |
|  |  | Proximal |  |
|  | Goal priority | Competing goals |  |
| 1. Memory, Attention and Decision processes | Memory |  |  |
|  | Attention | Attention control |  |
|  | Burn-out | Cognitive overload |  |
|  |  | Physical exhaustion |  |
|  | Decision making | Conflict |  |
|  |  | Appraisal of the decision |  |
| 1. Environmental context and resources | Environmental barriers |  |  |
|  | Environmental facilitators |  |  |
|  | Environmental stressors |  |  |
|  | Resources and materials |  |  |
|  | Organisational culture and climate |  |  |
|  | Salient events and critical incidents |  |  |
|  | Person environment interaction |  |  |
| 1. Social influence | Social support | Unconditional support |  |
|  | Group norm |  |  |
|  | Group conformity |  |  |
|  | Alienation | Within the health care system |  |
|  |  | Social |  |
|  | Power |  |  |
|  | Perceived intergroup conflict |  |  |
|  | Modelling |  |  |
| 1. Emotions | Anxiety |  |  |
|  | Stress |  |  |
|  | Depression |  |  |
|  | Instability of emotions | Emotional ‘roller coaster’ |  |
|  | Positive emotions |  |  |
| 1. Behavioural regulations | Self-monitoring |  |  |
|  | Action planning |  |  |
|  | Prompts and cues |  |  |
|  | Habitual behaviour |  |  |

*Coded themes represent barriers or/and facilitators of aspirin adherence as described by a cohort of non-adherent women participated in ANA qualitative study.*

* indicates themes related to informational needs. Those themes will be discussed in datil elsewhere.
