# Supplementary material for: Aspirin non-adherence in pregnant women at risk of preeclampsia (ANA): a qualitative study
Source: Health Psychol Behav Med. 2021 Aug 6;9(1):681–700. doi: 10.1080/21642850.2021.1951273 (PMC8354178; doi:10.1080/21642850.2021.1951273)
Supplement: Supplemental Material [file RHPB_A_1951273_SM7448.zip › Appendix 3 Table 2 (barriers).docx]

Appendix 3

Table 2 Examples of barriers to adherence

|  | Barrier | Quote | Quote | Quote | Quote |
| --- | --- | --- | --- | --- | --- |
| Knowledge  *An awareness of the existence of something* | Knowledge about the condition can be experiential, vicarious or theoretical | "Preeclampsia…I’ve heard of it. It is not where the placenta blocks the birth passage or something like that. I’ve heard of it, I never really… no…" (ANA 2) | "I kind of thought because my mum has had it and she come out the other side, we kind of said it’s 50/50. By the end I was that tired, I kind of thought: if I get that - I get that. It wasn’t a big deal" (ANA 12) | I think it was her (sister in law who had preeclampsia), she was unwell. They were monitoring the baby and he was always fine, but it was, cos she was just swelling up everywhere. So, it was complete bedrest, but everything she was, like everything was fine in the end. But you do like obviously that’s the only person I’ve ever heard of that’s been sort of taken in. (ANA 11) | "I don’t know. I never really got told much, just said I had to stay in so that they can keep an eye us and monitor and they I was told I am having a baby the next day. It was a bit shock to the system."(ANA 10) "I know pre-eclampsia can be monitored quite quickly. It can be controlled at the same time.”  “It normally happens in your first. Apparently, it’s quite common in your first pregnancy." (ANA 10) |
|  | Knowledge about medication | "Not really, I think initially it was my, it was the lack of information I had that you know." (ANA 5) | "I know it’s painkiller, pain relief, it gets rid of headache, it thins your blood. And I know if you are going to have a heart attack then stick one under your tongue. But when they prescribe these things, they don’t tell what they exactly for." (ANA 2) | "It’s a blood thinner isn’t it? I am assuming it keeps your heart pumping at the right level and your blood pressure down." (ANA 4) | “When you take your folic acid, you are taking the folic acid for the baby -you are not taking it for you... It’s drummed into you and everything you read… you are good mum if you take your vitamins sort of thing, whereas the discussion about aspirin is not, well… it didn’t feel like it was about that. It was about keeping the mum healthy and it’s the same with lots of medication (ANA14) |
|  | Task environment | "No, no one mentioned that it is available over the counter” (ANA 8) |  |  |  |
|  | Procedural knowledge (easier to take tablets) | "It would have been a lot easiest to take is as a tablet rather than soluble. Like if you are taking pregnancy vitamins, it would be a lot easier to take another one." (ANA 13) | "They gave me the one that you stir into the drink and because I was so sick it wasn’t a very good idea." (ANA 12) | "One thing I was never clear on. And I think asked the midwife, one thing I remember was not 100 % clear about was when I stopped taking it. Should I take it right up to the delivery or stop at some point when I was at term? That is the only thing I remember thinking about to do with the aspirin." (ANA 3) " | "I couldn’t keep anything down, tried eating something before it to see if that would help, may be because I was taking it wrong.” (ANA 10) |
|  | Necessity | "The aspirin in that nothing been….no symptoms would arise if I didn’t take it, kind of thing. Forgetting it didn’t lead to any incident’s kind of …or symptoms I suppose, which would make you to have the medicine." (ANA 3) | "It’s kind of like, well how to know this is making any difference and it’s weird doing something without knowing if it’s going to make any difference to you." (ANA 9) | "I hate putting anything into my body anyway. But when it like the vitamin D it was like, well, it’s going to benefit, if it benefits the little one then I’m quite happy to take it. But like I say, it was just the aspirin that was more questionable because like obviously vitamins are there to help you and like obviously in your system but aspirin like I say I don’t understand what benefits it has." (ANA 11) | "If people don’t feel like there are going to get a benefit from it especially a non-visible benefit from it, then what’s the point?" (ANA 14) |
| Skills *An ability or proficiency acquired through practice* | Never was good with medication | I always found taking medication quite difficult. Trying to think of a time where I had to take medication that wasn’t antibiotics... I think I had a back problems a few years ago and I was prescribed neurexin for it and I was supposed to take it in the morning and night and kept it for about 2 weeks. But I din't notice any difference, so I think I started taking that as and when I needed it or if I remember it. (ANA 13) | "I don’t really like taking them (tablets).”  "I’ve got anxiety and depression. I meant to take sertraline, but I won’t take it while I am pregnant cos I know it could affect the baby and the baby’s heart. I rather try maintaining all that and hormones…. "(ANA 10) |  |  |
| Social/ professional role and identity *A coherent set of behaviours and displayed professional qualities of an individual in a social or work setting* | Identity: not medication type | "I don’t mind taking medication, if I have to, but I would rather not." (ANA 6) | "I don’t like taking medicine, but I don’t even like taking paracetamol for headaches and stuff. No, it was fine I can take it I just, I don’t like my body relying on medicine. Like if I’ve got a headache, I don’t like taking paracetamol I’d rather my body gets rid of it on its own." (ANA 7) | "I am not really a tablet person" (ANA 10) | "I felt like, I felt like a pill bottle: if you shake me, I would rattle. Cause I don’t really take medication. If I have a headache, I don’t like go and take paracetamol straight away. I’d put up with it for a while before I took anything for it." (ANA 8) |
|  | Identity: unable to relate to the risk factors | "I wouldn’t say it’s got anything to do with your weight personally cos it can happen to anyone like the thinnest of people can have really high blood pressure and have blood clots. So, I think they put the stigma on people that are overweight and I don’t think, I assume it has some, you know thingy factor in it but I wouldn’t say it’s all to do with, cos everyone’s different." (ANA 11) | "All my check-ups with the midwife up to that point never showed any reason for concern to, there was nothing that was applied to me personally if that makes sense... it was that it wasn’t personal to (name), it wasn’t about me it, was your high risk your this, you know." (ANA 5) | "She (a midwife) told me I was going to get gestational diabetes and she was adamant I was going to be unwell and I didn’t like it. She said I could also develop pre-eclampsia. I was really angry. (ANA 13) | “I was shocked….I was so gobsmacked that to was given it (Aspirin) and I was perceived to be (at risk) because my sister had pre-eclampsia.” (ANA 4) |
| Beliefs about capabilities *Acceptance of the truth, reality, or validity about an ability, talent, or facility that a person can put to constructive use* | Low self-esteem/ low confidence/Self-efficacy | "Really difficult, I always found taking medication quite difficult." (ANA 13) | "I thought if I was given them, I was given them for a reason, but I am not the best with tablets. “I was more worried that if I asked too many questions that they would think I was maybes relapsing. I kept my worries to myself." (ANA 4) | "I’m no good at taking tablets even the folic acid tablets and stuff I didn’t really take them. Yeah, I think I’m just generally bad at taking tablets."  (ANA 7) |  |
|  | External locus of control | "I think like with anything; anyone can get it I don’t think you have to be a certain way I don’t think you have to be a certain age. I think if you’re gonna get it - you gonna get it. It doesn’t matter what shape you are what size you are it’s, if you’re gonna get it you’ll get it". (ANA 11) | "I didn’t worry about anything because at that point I gotten into the attitude of if something happens - it’s going to happen anyway, and I think it was a big part of being able to relax and keep my mind at ease. It was going to be fine and whatever happened, happened. You can’t control things and it’s not like you can do everything and if you get pre-eclampsia you get pre-eclampsia. It happens. There is no way of stopping anything, so in my head I knew I was totally fine." (ANA 13) | "Just potluck really." (ANA 7) |  |
| Optimism *The confidence that things will happen for the best or that desired goals will be attained* | Unrealistic optimism | "Well it was just to be honest with you it was just completely focused on the baby I didn’t think that I would (have preeclampsia), there would be anything wrong with me because my mum didn’t have anything, any problems or anything like that. My sister didn’t have anything on her second one she had a bit of spike in her blood pressure but that went down so there wasn’t any, you know we didn’t have any ongoing issues or anything like that. I wasn’t worried. Look at me, I don’t have any symptoms" (ANA 5) | "I never thought you could get it twice." (ANA 10) | I think I had good feeling that it was just never going to happen. I just didn’t think I would ever have pre-eclampsia... It was never something I really worried about and I don’t I ever thought my chances of pre-eclampsia are really high. " (ANA 12) |  |
| Beliefs about consequences *Acceptance of the truth, reality, or validity about outcomes of a behaviour in a given situation* | Beliefs about consequences of taking medication | "I wasn’t actually, to be honest that’s why I wasn’t wanting to take them - in case like she got addicted. I was concerned about heart defect or something, but I didn’t actually question it. " ANA 8  “So, do I just find out at a scan that something is not right? Is it down to the aspirin, is it not down to the aspirin? But again, those are the questions I didn’t want to ask. I think I was too frightened to ask." (ANA 4) | "The aspirin knocked me very sleepy, knocked us with headaches made us feel sick a lot. It made me very sick." ANA 10 | "I don’t know, I didn’t feel great on it, it didn’t make us feel any better than I, cos I had a lot of like sickness and stuff. But when it comes to taking it, I still think that had something to do with my bleed, I say it does." ANA 11 | "I think one risk I didn’t really get to grips with would be the increased risks of like in terms of haemorrhage and bleeding and stuff if you are on aspirin. I don’t know how…much about that you know a valid risk because a lots of women general discussion within your community is about blood loss and how much blood you lose. I think it’s a massive thing. Are you going to bleed to death, or are you going to be anaemic afterwards, or are you gonna have to have a transfusion and I don’t remember anybody saying anything about that at all. But as a lay person, I can image if I take that aspirin does that mean I am going to bleed more when I have my baby? And that might frighten you to not to take it, because culturally you are so frightened whereas as blood pressure is hidden -you can’t see it and you can’t see the effects that might be having on your baby. You can’t see any of these, but you can visibly see a lot of blood, so I think more people think about that more." (ANA 14) |
| Reinforcement I*ncreasing the probability of a response by arranging a dependent relationship, or contingency, between the response and a given stimulus* | Contingencies | "So, if they get it middle of the day most of the time there’s no point taking the aspirin cos they’ll probably have been sick and they lose the, bring it up." (ANA 5) | "If I remembered to take them, I took it if I didn’t, I didn’t" ANA 7 | "I think you need to make it more personal like by asking the individuals do they have sickness. If they don’t suffer from sickness, then that’s fine - they can create whatever routine. But if they do suffer from sickness you have to be really careful about it because the flip side is if she’s taken one and she’s sick the likelihood of her taking another one is nil because she’s not going to want to risk having taking a hundred and fifty milligrams when the dosage is seventy five." (ANA 5) |  |
|  | Lack of reinforcement/negative reinforcement | " When I told her (a friend who is a health care professional) that I’d obviously stopped taking it, she said you know you’re body better than anyone, she said, and if you’re not comfortable, she says, I would always say don’t take anything that you’re not comfortable taking." (ANA 8)  "He (participant’s father) said: ‘I didn’t want to say anything at the time’, he went, ‘but I don’t know why they would give you aspirin in pregnancy anyway, it’s not something that I would think that you would tell a pregnant woman to take.’." (ANA 8) | "And when I told her, obviously my midwife, that I’d stopped and she said: ‘well that’s entirely up to you, if you feel that it’s not right for you’, she says then it’s just something obviously that they recommend that you do when you’re a higher risk. But she said; ‘if you don’t want to take it, then that’s entirely up to you’. And I stopped and I was fine." ANA 11 "He (partner) said: ‘only you can make the choice. If you’re not happy taking it, then don’t take it. There’s plenty of women that go through pregnancies and don’t take it.’ So yeah, he was supportive and just said, he says I just support whatever you want to do." (ANA 11) | "I didn’t feel like asking. By this point I wasn’t seeing anybody, so I didn’t who to ask I suppose. Feeling that midwife… there should be somebody there to discuss it more in depth with, I think. " ANA 4 | "It's not presented…the midwives didn’t want to take any responsibility over that. They are quite happy to push vitamins folic acid. But whereas if you had a midwife saying this is good for your baby and I am going to push this. And your community midwives that you had built a relationship with then you might see it slightly differently and take it differently. But it was very…in my experience very much a, that’s a hospital decision ... " ANA 14 |
| Intentions *A conscious decision to perform a behaviour or a resolve to act in a certain way* |  | "I didn’t really want to take it. Obviously, I am not one for taking medication and things that." ANA 6 | "I didn’t want to take them. I don’t need them. I went home and read the leaflet and the side effects." ANA 4 | "No, I didn’t get it (the prescription). I forgot to be fair. I was a bit a wary to take it because didn’t take it with my other, 3 so I was like: "why do I have to take it?!”  “I haven’t even got the prescription yet. I kept saying I’ll get it tomorrow. I didn’t think I need it, so I didn’t take it.’ ANA 1 | "So initially you’d say yeah okay, then but kind of like go home you sit downright okay what am I doing I’m taking this right what does it say, what is it for, what is, what does it do." (ANA 5) |
| Goal  *Mental representations of outcomes or end states that an individual wants to achieve* | Priority | "The sickness was very drowning on us all the way through the pregnancy. It was just horrible, really, really tiring. I was finding that I couldn’t keep anything down. when I had something to eat and 10 mins later, I am bringing it back up. The fluid I found hard to keep down at first then I managed to maintain fluids a lot and managed to keep that down." ANA 10 | "I knew it was important to take aspirin, but some days it was so much more important to get anti-sickness tablets down us with a bit of water because some days I couldn’t even drink water." ANA 12 | "Once that ran out it took us a few weeks to buy another box because it wasn’t something I was thinking of. If I was out and I spotted them in the supermarket then I bought them. It wasn’t my top priority, if you know what I mean." ANA 2 |  |
| Memory attention and decision making  *The ability to retain information, focus selectively on aspects of the environment and choo*se between two or more alternatives | Memory | "I don’t take it take it all the time because I forget. I try to remember to take it all the time just once a day, but I do forget." ANA 2 | "Again, it was purely just I would get out and about and forget to take it. It wasn’t really a concern or worry about it." ANA 9 | "Yeah, my forgetfulness like was constant thought me pregnancy, it still hasn’t corrected itself. I just called it baby brain." ANA 8 | "And I think just a general brain fog in pregnancy as well and made me a bit stupid a lot of the time, especially towards the end. I do think it had an impact on me." ANA 3 |
|  | Attentional control | " I feel like sometimes I got distracted because sometimes your mind starts to wander a bit, you know like if I was just sitting and then I’d start, cos I used to worry so much after my bleed like so much. Then I’d forget what I had to do, or I’d forget that I had to go like and meet him from school. It’s like you just go blank, and I’m thinking of so many other things it was mostly pregnancy related and I would have to go, right, what am I doing again." (ANA 11) | "Probably a little bit worse because when I am pregnant, I really got no attention span to anybody else” ANA 10 |  |  |
|  | Cognitive overload and tiredness | "No, every day I have a cup of tea, and that’s why I think I try and focus on taking medication at that time, but then anything where you have taken more than one a day I sort of forget, so I don’t have a reason to remember to take it" (ANA 13) | "So we tried to put a routines in place but I think the tiredness associated with pregnancy, just the fact that you are a bit out of your routine, quite a lot of the time in pregnancy because your body is doing different things, you are feeling things, I think that kind of threw me out." (ANA 3) | "It was more to the end of my pregnancy when I started missing them. I was getting too tired and I was just going to bed and forgetting." (ANA 6) |  |
|  | Cognitive overload/ information | "I think it was just us taking in so much information. I hadn’t digested that information to then go, right, okay, so, you know I’ve said okay, but what is it that I’ve said okay to? What is the impact that that’s going to have on me? What’s it going to do to the baby? And you get the aspirin, then you read the precautions. So, you don’t have time to process that information because there’s just so much being told to you, and they don’t tell you that you know. Cos when people open up the boxes when you’re pregnant the first thing you do is check is it suitable for pregnant women, you know what I mean? And it doesn’t even say seventy-five milligrams is safe or anything like, you know, which they won’t because they’re not going to put themselves in that position. But that type of information you don’t have time to kind of like go through it all, because you don’t know what that’s going to say." (ANA 5) | "Obviously when I was pregnant, I’ve read lots of stuff, but you get bored of reading stuff and it’s too much. And it’s like once you read the first page you are like, oh my goodness, I am not reading all that, and you push it all to one side and me personally I through: ‘I am not reading all that’. " (ANA2) | "I don’t know, you know having that talk during that meetings that you have with the consultant if you imagine how many things get given to them the pregnancy books for example they give you a book that big, how many people have read it, you know what I mean? " "You don’t have time to, process that information because there’s just so much being told to you." (ANA 5) |  |
| Environmental context and resources *Any circumstance of a person's situation or environment that discourages or encourages the development of skills and abilities, independence, social competence, and adaptive behaviour* | Obtaining medication | "I got the prescription on the day I had my 12 weeks scan and I took it to the pharmacy and they said it wouldn’t be available until the next day and at that point I lived in xxx and I didn’t have any way to get there and I never ended up picking it up honestly... until…I can’t remember. I think it was after my 20 week and I started the aspirin a couple of days before my 20-week scan so that was my reason of not taking it, up until 20 weeks." (ANA 13) | "I’ve got the prescription for aspirin. But the hospital pharmacist said we have to wait for like an hour and half, so I said I am not waiting for that length of time. So, I didn’t get the prescription at the hospital." (ANA 2) | "I did mention something about buying it. Whoever I spoken to said that you couldn’t buy that dosage over the counter. You can buy aspirin, but not that dosage." (ANA 12) | "I think I was as given the prescription, but I think ended up buying it over the counter for some reason, but I can’t remember the reason it was because I was that efficient and took the prescription the pharmacy in the hospital or vice versa but I remember I ended up buying it over the counter because the GP didn’t know I had a prescription, but I just bought it over the counter throughout the pregnancy because I couldn’t be bothered to pick the prescription anywhere. (ANA 09) |
|  | Replenishing medication supply | "I missed a couple of days of taking my aspirin because (when I) asked the doctor surgery for it and they said it hasn’t been sent through, so it’s not in your file and they have to contact the (hospital name)." (ANA 4) | "I just went to the GP’s and told them that I had ran out and asked them if I could have some more on a repeat prescription and they told us I couldn’t because I was pregnant." (ANA 7) | "(Replenishing medication) it was really difficult. I took it to the doctors, but they didn’t put it on the prescription, so I then I had to ring up my hospital too and get my medication cos the doctors didn’t have it on their system. This happened a couple of times to be honest and I did go without medication for a few days because like it was so much of a hassle to try and get it." (ANA 8) |  |
|  | Organisational culture and climate | "It’s not presented…the midwives didn’t want to take any responsibility over that. They are quite happy to push vitamins folic acid. But whereas if you had a midwife saying this is good for your baby and I am going to push this. And your community midwives that you had built a relationship with then you might see it slightly differently and take it differently. But it was very…in my experience very much a, that’s a hospital decision ... very separate. Considering there was one person being looked after and I am same person whether I am in the hospital or with a community midwife there were very different agendas, very different agendas. " (ANA 14) | "The doctors, I have to admit, were a bit like coming into the room, sit down, look through your file: “right, right, you’ve done this, right, and everything seem to be ok, see you next time”. There wasn’t a lot of interaction with the consultants. I didn’t feel like, you know, sometimes your GP you get to know them, they are friendly, and you have a chat as well as talking about your problems and your health. But with the consultants at the hospital it was just like… it’s hard to describe come in, hello, see you later, next, come in, see you later, buy, come in…. It wasn’t like full understanding; they were not giving full explanation. (ANA 2) | "Because my GP consist of 5 different midwives and I have issues dealing with people who I don’t know, so I couldn’t build any kind of relationship because I didn’t see the same one and I do have issues with things like that. " (ANA 4) |  |
|  | Resources (in a context of fiscal, human resources, or time) | "Yeah because I mean for me it worked after the second time (appointment), the first (appointment) I was: "people are crazy", you know what I mean? Because I wasn’t convinced you have to (talking about taking aspirin)" (ANA 5) | “But then everybody is on time scale, I was arguing in my head, you got some many people to see within certain period of time, so then if you are running late… I am aware of that.” (ANA 4) |  |  |
|  | Person x environment interaction/ passive | "I think at the time I didn’t ask the question when I was given it because I was so gobsmacked that to was given it and I was perceived to be because my sister had pre-eclampsia." "I didn’t ask enough questions and I didn’t look into it." ANA 4 | "I felt more comfortable with her (midwife) I got on really well with her she was very down to earth. She wasn’t one of these as I call textbook professionals where it’s not coming from a personal side, it’s coming from a textbook. Like I wanted, I like people who advise you through, you know, like through life, you know, this is what they recommend, but it’s whatever you feel comfortable with sort of thing. Where you get some healthcare professionals doing this is what you must do, and you cannot like deter from the track this is what you’ve got to stick too. So, I felt a very sort of, she was very comforting she was sort of very, just on like she was like an everyday person someone that you enjoy sitting talking to and you can ask her absolutely anything and you would get a straight answer. It was just straight to the point and I prefer things like that. (ANA 11) | "I feel like if… if somebody is not telling me the right answer, I’ll go and ask somebody else. I know I would get it answer eventually. I’d would get a second opinion if I didn’t actually trust somebody." (ANA 8) |  |
|  | Salient events and critical incidence | "I would always try to make sure to keep on top of my meds, but I was on 4 different anti-sickness tablets on top of my vitamin D and Folic acid, and aspirin and there were other things by the end. Sometimes I didn’t take it regularly because I was so unwell and sick. I would either forget or I would take it and I was sick. I couldn’t eat anything through the pregnancy." ANA 12 | "I ended up having OC in my pregnancy which was very nerve racking because it could eventually harm my baby. I was constantly thinking something is going to go wrong. Is she going to be born stillborn? I had gall bladder stone so I was in quite a lot of pain with that and my back pain was horrendous. I am still suffering; I was just constantly in pain. It was just horrible." (ANA 10) | I had a massive bleed at fourteen weeks, yeah fourteen weeks or was it thirteen weeks? I had a huge bleed and I was at work. It was awful. It was diagnosed as a threatened miscarriage, scary." (ANA 11) | "I lost my sister when I was 8 weeks pregnant and that was a very strange one because none of my family knew, so dealing with that was a bit intense. I think the worst things that could happen, we also had to move out of our flat" (ANA 13) |
| Social influence *Those interpersonal processes that can cause individuals to change their thoughts, feelings, or behaviours* | Social pressure | "My husband wasn’t very keen for us to be taking them either." " I did discuss with my sister and she said she didn’t feel any benefit of it, obviously she still had the pre-eclampsia, but she was quite convinced that some of my nephews’ problems were down to taking the aspirin. He is 17 now but he has just been diagnosed in the last 3 or 4 years."(ANA 4) | " She (my sister) wasn’t happy, but she doesn’t take medication. She is very much like a natural medication kind of person and when said I was taking aspirin, she said I can’t take it, but I said I am going to do it." (ANA 13) | "And like nobody…there is nobody pushing me to do any of this. There is nobody saying you’ve got to do this. I don’t have anybody in life that saying- you need to this… pushing me to do anything. I don’t have any of that going on, it’s all me. I make the decision for me. When it came to the aspirin, I obviously discussed it with my husband and my mum, but to be perfectly honest neither of them would change… I would listen to what the medical professional had to say and then as an individual I probably would have made the decision. They didn’t sway me either way." (ANA 14) |  |
|  | Group conformity | "She (a friend) told me, she hadn’t really been taking them, only in hospital. So, she didn’t know the benefits of taking them." (ANA 8) | "Yes, one of me friends, she was prescribed aspirin. But I don’t think she took it either. I think she was prescribed it because of her weight and because she smokes." (ANA 1) |  |  |
|  | Isolation/Alienation | "yeah, they’ve (friends) never been through it with pre-eclampsia so they didn’t understand why I was taking it." (ANA 6) | "No, she was completely low risk. I can’t remember what she said to be honest. I definitely discussed it but I probably didn’t discuss it at length because she was sort of like... it’s difficult discussing things with another pregnant women when they’ve got their own agenda and own worries and their…. So I would have definitely talked to her about it but I don’t think anything she said would have made any difference because I would have been like you got your things to think about and your own worries so… probably because she is low risk and she doesn’t have that decision to make, so she doesn’t need to invest much thought into it. " (ANA 14) | "No, not really, they would have, if I brought it up to them but I didn’t really bring it up to them. No, no I don’t like to worry people." (ANA 7) |  |
|  | Dissatisfaction/Lack of Trust | "I do realise nothing was explained to me and I wasn’t quite listened to in terms of risks. No one said anything apart from it was all focussed on weight really which wasn’t great." (ANA 13) | "I knew at that point I was high risk, but I wasn’t monitored any more than a normal pregnancy. Do I don’t know how people can put you in high risk bracket for no extra monitoring. Just go away and take this (aspirin).”(ANA 4) | "And I think they need to be more honest with that because obviously they must have more than a few people coming back saying that they don’t feel right on taking it (aspirin). I’ve always believed that, that was the reason for the bleed it’s not co-incidental that you know, nine, ten days after I started taking it that I bled, to when I stopped taking it that the bleeding stops. They’ll (medical professionals) never agree, they’re never going be, that’s what I say like when they’re sitting there behind their computers and their books they don’t, they’re not honest enough. Where they should just be honest where they cannot like refer to a page in a book or like a script because this is what should be happening." (ANA 11) |  |
|  | Intergroup conflict (perceived)/ conflicting information | "I got a prescription of it from the (hospital name) and then after that I had to go down to my doctors and get it. But they had to ring the (hospital name) to make sure that I was allowed it, cos you’re not allowed to take it during pregnancy it’s not recommended." "No, cos I kind of knew that you weren’t cos it’s written on the box. So, I knew you weren’t allowed to take it" (ANA 7) | "Do you know there’s obviously some kind of risk as to why, if it was that good for you if you’re high risk why would the pharmacies like not be willing to sell you aspirin when you’re pregnant? So, there’s some kind of, do you know what I mean?" (ANA 11) | I did read the side effects. I am sure it did say they weren’t sure whether if it did cause problems during pregnancy. (ANA 4) | "..when people open up the boxes when you’re pregnant the first thing you do is check is it suitable for pregnant women, you know what I mean? And it doesn’t even say seventy-five milligrams is safe". (ANA 5) |
|  | Power | "When you see the consultant, you feel a bit like intimidated, like being back at school, headmaster sort of feeling. They weren’t chatty, they were like I say: everything looks fine, we put you in for another appointment in 4 weeks’ time. Have a nice day. That was it. Like I said the big bosses, the consultants you were a bit intimidated. I suppose they are not there to be friendly, they are there to do a job but at least you want to go in not feeling nervous." (ANA 2) | "I think it was a bit more relaxed with the group and the midwife because it informal and just relaxed and you didn’t feel like you were going to get told off by the doctor for not taking your medication or forgetting it in the past." (ANA 8) | "He (partner) said: ‘I’m surprised you didn’t say anything’, cos I’m quite vocal where I just say it how it is. I said: ‘well, no, because I’m in like her environment’, do you know what I mean? It does feel like, it’s like regimental...Well that’s it because you’re in their domain it’s like they’re in control, if you know what I mean? And you do feel like you’re getting spoken down to like: " I know better, this is what you should do". " (ANA 11) |  |
| Emotions *A complex reaction pattern, involving experiential, behavioural, and physiological elements, by which the individual attempts to deal with a personally significant matter or event* | Anxiety | "Definitely, I was anxious, very anxious. I don’t mean it in a kind of… it was like a low-level anxiety; I am not saying I was cripplingly anxious constantly. I just had a low-level constant anxiety running through it, made me distracted I think." (ANA 3) | "I had my low days; I think with any does. When I was having my low days, I would try and think of something else like playing with my daughter, try and go out, try and cheer myself up. But someday I did really struggle, have my blanket out sit on the sofa and would have my little girl next to us and cuddle up… which my little girl quite likes doing. It can get a bit draining and get too much especially when you are pregnant, and you got to deal with your hormones, fear and anxiety going out by yourself. it’s quiet nerve racking sometimes." (ANA 10) | "I think I was more; I think it’s more worry takes over: is everything okay, do I really want to know if something’s wrong? Am I going to worry for the next week before I can have another appointment? " (ANA 11) |  |
| Behaviour regulations  *Anything aimed at managing or changing objectively observed or measured actions* | Denial | "It wasn’t something I was thinking about. I don’t know, I was thinking about being happy because I was pregnant rather than taking about tablets." (ANA 2) | "So, it’s just like there’s so much information there and if you’re a person that normally stresses that would just, that would send you over the edge. So, I don’t know I wouldn’t, I wouldn’t want to read all of the leaflets that are out there." (ANA 5) | "I am just scared of answers, I think it was just that, scared for the answer to be honest." (ANA 8) |  |
|  | No habit | "I think, perhaps, I am not in very much in the habit of taking things. So sometimes a day would go past and I would realise I hadn’t taken it. It was just that the day had gone pas and in all its business and general kind of chaos, sometimes, with my eldest and I just hadn’t got to the point where I did manage to take it. Perhaps simply because I am not in a habit of taking it..." (ANA 3) | "it was a little bit challenging because it was a whole new thing, I was just used to one routine with my routine." (ANA 10) | "More by the end of the pregnancy I was really on top of it and I think that’s when its’ more worrying for pre-eclampsia, in your last trimester." (ANA 12) |  |
