# Supplementary material for: Aspirin non-adherence in pregnant women at risk of preeclampsia (ANA): a qualitative study
Source: Health Psychol Behav Med. 2021 Aug 6;9(1):681–700. doi: 10.1080/21642850.2021.1951273 (PMC8354178; doi:10.1080/21642850.2021.1951273)
Supplement: Supplemental Material [file RHPB_A_1951273_SM7448.zip › Appendix 3 Table 3 (facilitators).docx]

Appendix 3

Table 3 Examples of facilitators of adherence

|  | Facilitators | Quote | Quote | Quote | Quote |
| --- | --- | --- | --- | --- | --- |
| Knowledge | experiential knowledge/  procedural knowledge (relates to the condition or the medication) | "I explained the circumstances of my previous pregnancy and delivery which happened in X country. I was induced for that delivery and my eldest ended up being small for the dates. He was a lot smaller than expected to be. And some of the reasons why he was induced pointed to probably preeclampsia – my blood pressure was raised. " (ANA 3) | "I could remember going into the hospital, but I couldn’t remember anything after then. When I had come around that when I got told I had an emergency C-section." (ANA 6) | "The tablet was much better, so I rang me GP and me GP prescribed the tablet version, so I took the tablet version instead. Because drinking the drink wasn’t very nice and it’s was going to knock me more sick, so I just thought I don’t really want that." (ANA 12) |  |
| Skills | Taking medication already | "To be honest, I didn’t mind, because from the minute I found out I was pregnant I started taking the "Pregnicare" tablets.” (ANA 5) | "Well I took vitamin D anyway I had extra supplements for that so I’ve just, I took it alongside that every morning. So, it wasn’t a major obstacle, do you know what I mean it wasn’t a hindrance to have aspirin cos I was already taking my vitamin D, so it was just an extra thing to take." (ANA 11) | "I take the contraception pill every night before I go to bed." (ANA 6) |  |
| Social/ professional role and identity |  | "So, then because you feel having these extra attention probably pushes even more to look after myself in other ways and take responsibility for my own health in other ways, not just taking my medication, and other ways in terms of weight and exercise and following other guidance just to try and look after myself as best as possible. And then the whole team around you can look after you." (ANA 14) |  |  |  |
| Beliefs about capabilities | Self-esteem | "I am the total opposite I am quite head strong when it comes to medical professional" (ANA 13) | "I wanted to try and if I eat healthier and exercise, I will feel better, it will make me feel better and then it’s "mind over matter". I did feel better, I went swimming, had a good diet, I went to yoga, so I enjoyed all these things. I didn’t enjoy them the first time, I enjoyed them the second time and that makes you feel good as a pregnant woman to do all these things and think I can go through all these things and look at me swimming when I am 30 odd weeks pregnant, that makes you feel good. Gives you that rush that you’ve achieved something, and I went to yoga right up to the week I give birth, so again it’s the same thing, it’s a positive thing that you are still doing this and still being active." (ANA 14) |  |  |
|  | Self-efficacy | "Once I got on top of it, I probably felt loads better, I was taking every meds I was supposed to, and there were no worries anymore." (ANA 12) | "I would say it’s really up to yourself it would be, it’s only yourself who can like sort of prevent medical things from going on and everyone else is just there for general support. But I would say it’s down to me to prevent like to help prevent any medical conditions. I wouldn’t say anyone, you know you’ve got the medical side that will help you and can guide you, but I would say it’s down to yourself to stop any medical conditions." (ANA 11) | "Once I got into a routine, I was quite capable to keep up with this" (ANA 10) |  |
| Beliefs about consequences | Positive beliefs about medication | "No, I didn’t have any concerns about it. I was happy to be prescribed something that would hopefully prevent any problems with the placenta." (ANA 3) | "I do really worry when it comes to tablets in pregnancy. But I know if it’s’ going to save my baby’s life then I will do it." ANA 10 | "You have to think about your actions, haven’t you? If I hadn’t taken the aspirin what’s to say I wouldn’t have more complication than what I had come the end when is stopped taken the aspirin. Is there a consequence because I stopped the aspirin that’s why I got the problems"? (ANA 4) |  |
| Reinforcement | Sanctions | "Until I found out the complication of not having the aspirin, I wasn’t really bothered about that. So, I did leave it about a week you know…" (ANA 8) | "I think they said there could be risk if I didn’t take it." (ANA 6) |  |  |
| Intentions |  | "Cause you know, these doctors know best, so you listen to them and you try your best to follow them. It’s the best thing for ya. When you haven’t got their advice you are just all over the place, aren’t you." (ANA 2) | "No, I never really had any doubts on it because my mum took it. It had already been prescribed by the consultant at the hospital I knew I needed it. I had the discussion with her, so as soon he had a look through my maternity notes, he is doctor, so he understands what’s written on there. Him looking at my maternity notes was a little reassuring in that sense that he read through it and he knows exactly what I needed for. So, I never really had any doubts about it. Kind of thought its something I need, so I am going to take it." (ANA 12) |  |  |
| Goals | Facilitating goals | "Just hoping that baby would be ok, to be honest." (ANA 8) | "I always worry that, that was what was going to happen and that…that the same thing would happen basically. I was worried that my blood pressure would go up and I was worried that the birth would be the similar thing. I was worried that I would be all in a room full of monitors and doctors." (ANA 14) | "Some people are not good at it, but you have another reason there’s a baby involved here so you know, you have to kind of get in to the." (ANA 5) |  |
| Environ. context and resources | Person x environment interaction /able to navigate the system/ active | "GP didn’t know I had a prescription, but I just bought it over the counter throughout the pregnancy, because I couldn’t be bothered to pick the prescription anywhere." (ANA 9) | "When you’re high risk, you have more appointments and that’s when I’d spoke to her and I said I need to speak to somebody I can’t remember exactly on the phone conversation. I think it was my own midwife, because if I called direct to the hospital, I wouldn’t think they would put me through I think it was my own midwife." (ANA 5) |  |  |
| Social influence | Modelling | "My husband takes medication regularly; he has to take medication daily. It’s just like clockwise, he gets up and takes his medication and that’s it, this is what he does. " (ANA 3) | "And my parents are diabetic, so I know the routine I know what it should be what it shouldn’t be and things like that so, I think you just develop a routine." (ANA 5) | "My mum takes lots of medication, she has rheumatoid arthritis, she is epileptic, and she is diabetic, she has glaucoma, she is deaf. She is on aspirin as well." (ANA 2) |  |
|  | Support | "He (partner) was more for it than I really was. He was there when it all happened with my first with pre-eclampsia. He was more for it to take it than anything." (ANA 6) | "My nana said if I didn’t take them, she is going to kicks us up the butt. She was very supportive" (ANA 10) | " So yeah, he (partner) was supportive and just said, he says I just support whatever you want to do." (ANA 11) |  |
|  | Trust | "If I am given something there is a reason why I am being given the medication. Its not just because doctors don’t hand out medication willy-nilly. There is a reason and when you think logically about it." (ANA 4) |  |  |  |
| Behaviour regulations | Habit | "Easy, once got into it, cos it was the water-soluble aspirin. It was pretty much similar routine to what I have now. I would get up in the morning and have my cup of tea with breakfast and then get a cup of water and do the aspirin and would take them all together. I remembered most days I think I tried to take them at the same time every day, so I like to make it routine" (ANA 13) | "I was doing it the same (with aspirin). On a night-time I would take before I went to bed. I take the contraception pill every night before I go to bed so it’s just getting in the habit of taking it." (ANA 6) | "Yeah once I got into a routine, I was quite capable to keep up with this" (ANA 10) | I didn’t miss anything because I would get up in the morning sit down have my cup of tea and prepare my medication and breakfast." ANA 13 |
|  | Action planning | "We had a pill box by 27 weeks because I felt I was forgetting all of the time." (ANA 12) | "Yeah, beside me bed, that’s where I started leaving in the end, cos I would get up and take and go and sort my daughter out. (ANA 8) | So, I used to take that every day and I had my routine it was just by my bedside right before I went to bed, I would take it or early in the morning when I woke up I take it. So it wasn’t, the routine wasn’t an issue because I was taking the extra supplements anyways." (ANA 5) |  |
|  | Prompts and cues | "When I was taking the medication through the pregnancy, I had to put my tablets in the kitchen on top my microwave so when I walked into the kitchen, I’ve seen them, and I knew I had to take them. So that’s done it, I had put things where I could see. Any appointments, I put on kitchen cupboards." (ANA 2) | "It really was just that I have opened that cupboard that morning, so I have seen so I have taken it." (ANA 9) |  |  |
|  | Monitoring | "I had a little white board thing on my fridge, and I would have my medication wrote down and tick them on the days I took it os I knew I had to take it, the time I was going to take it. It worked once I got used to it." (ANA 10) | "He brought is a notebook to write down what time and what I had took so that I was on top of everything." (ANA 12) |  |  |
