# Supplementary material for: Aspirin non-adherence in pregnant women at risk of preeclampsia (ANA): a qualitative study
Source: Health Psychol Behav Med. 2021 Aug 6;9(1):681–700. doi: 10.1080/21642850.2021.1951273 (PMC8354178; doi:10.1080/21642850.2021.1951273)
Supplement: Supplemental Material [file RHPB_A_1951273_SM7448.zip › Appendix_1_Interview_topic_guide.docx]

**The “ANA” study**

**Understanding AspiriNA dherence in pregnant women at risk of preeclampsia:**

**A Qualitative study**

**Interview Topic Guide**

**Introduction**

- Researcher thanks participant for their time to attend the interview.
- Researcher explains the purpose of the interview.
- Use of a dictaphone is explained to participant and instructions provided to switch off the device should the participant wish to do so.
- Researcher assures confidentiality.
- Provide participant with an opportunity to pose questions about the project. Inform participant that the interviewer is unable to answer any medical questions.
- Explain that the interview can be ended or postponed at any time.
- Informed written consent obtained.

**Background information**

| **Age** |  |
| --- | --- |
| **Ethnicity** |  |
| **Professional background** |  |
| **Risk factors** |  |
| **Treatment for chronic illnesses/ comorbidities** |  |
| **Parity** |  |
| **Smoking status** |  |

**I understand that you were considered to have higher risk pregnancy this time…..** How did it go for you this time?

| **Questions and prompts** | **Domain** |
| --- | --- |
| 1. How were you **feeling** in your last pregnancy: *Were you able to enjoy your day-to-day activities?* | **Emotions** |
| 2. Thinking back to the time of your last pregnancy what were your goals and priorities during your last pregnancy?  3. In terms of goals and priorities you set during your pregnancy has your focus changed over time? | **Goals and Priorities** |
| 4. What did you knew about the **risks** in your pregnancy?  *Prompts: Could you please tell me why there were risks?*  *At risk of what?*  *Who told you about the risk?* | **Knowledge** |
| 5. What did you **knew** **about preeclampsia** when you became pregnant? | **Knowledge/skills** |
| 6. Looking back into the time when you were told about your risk of preeclampsia, were you aware of any **preventive treatments available**?  *Prompts*: *What are they?*  *Who told you that?*  *What this treatment does (how, dose, when, mechanism)?* | **Knowledge/skills**  **(Coping)** |
| 7. What do you know about the **complications** of preeclampsia?  *Prompts:* *Who told you about it?* | **Knowledge**  **Beliefs about consequences** |
| 8. What do you know about **advantages** of the aspirin treatment proposed? |  |
| 9. What do you know about **disadvantages** of the aspirin treatment? |  |
| 10. Have you had any specific **worries** during your pregnancy in relation to taking aspirin?  *Prompt:*  *Were these worries about the impact on your health?*  *Were these worries about the impact on your baby’s health?*  *Comparing worries about negative* ***effects*** ***of the medication*** *and* ***complications brought by PE,*** *what did you thought weighted more in your* ***decision*** *to take/not to take aspirin?* | **Beliefs about consequences**  **Decision making process** |
| 11. Could you recall how did you **feel** when you were pregnant about your **odds (likelihood) of developing** preeclampsia? | **Optimism** |
| 12. When you had a conversation with the doctor do you remember thinking about the **necessity/ importance of taking the aspirin**? | **Decision making process** |
| 13. W**hat were your thoughts** when you were trying to decide whether you should take the aspirin as prescribed or not? | **Decision making process** |
| 14. What was your immediate **intention on the aspirin intake?** | **Intentions** |
| 15. Has this intention **changed** over time? | **Intentions** |
| 16. Considering the period of your pregnancy and the time when you were taking the aspirin, did you at any **stage** felt differently about it? When was that? Could you describe this? |  |
| 16. What did your **family thought** about taking aspirin during your pregnancy?  17. What did your **friends thought** about taking aspirin during pregnancy? | **Social influence** |
| 17. Could you please give me some examples when you, unwillingly, forgot or missed taking the aspirin as prescribed? Was this a recurrent event?  Prompt: *Would you say that this is something that happened often?*  *What other reasons lead you not to take the aspirin as prescribed?* | **Intentions**  **Memory** |
| 18. Could you please tell me about your **experience with aspirin**?  *Prompts: How was it for you taking it every day?*  *Were you told how to take it?*  *Were you* ***prescribed*** *aspirin?*  ***Who*** ***advised/prescribed*** *you to take aspirin?*  *Do you* ***remember what dose and how often*** *were you advised to take?* | **Skills**  **Attention**  **Environment**  **Resources** |
| 19. Could you please tell me about any times where you felt it was **difficult for you to concentrate** during your pregnancy? Any impact on your adherence to aspirin intake as prescribed? | **Memory**  **Attention** |
| 20. Were you taking any other **prescription medication** at the time? | **Skills** |
| 21. Could you tell me how it was for you **to add taking aspirin into your routine?** | **Environment** |
| 22. Any there any **difficulties** in doing it? Did you find taking aspirin **challenging**? | **Beliefs about capability** |
| 23. How easy was it to **replenish** your aspirin supply?  *Prompts: Could you please tell where you were getting your aspirin from? Was it difficult to get a repeat prescription? If you bought it over the counter, was that expensive?*  *When you got aspirin in the pharmacy or from GP, did it ever happen that the person that made a comment? What type of comment?*  *What did you thought this person was trying to say to you? How did that make you feel in terms of taking the aspirin during pregnancy?* | **Resources**  **Social influence**  **Reinforcement** |
| 24. During the pregnancy, did you feel that the treatment you were having had a **positive effect** on you or your baby’s health? | **Optimism** |
| 25. What motivated you to take aspirin/ not take aspirin? | **Reinforcement** |
| 26. Could you tell me of any occasions you were supported by others with the aspirin intake? Who has supported you and how? | **Positive reinforcement** |
| 27. What strategies have you used **to support you in taking the aspirin as prescribed?**  Eg dairy, phone alerts, your family members reminding you… | **Social support**  **Resources** |
| 28. Can you tell me a little bit about the **relationships** with different **medical staff** you had caring for you during your last pregnancy?  *Prompt: Did you feel you had a good relationship between the health care staff caring for you during your pregnancy?* | **Environmental context and resources** |
| 29. Looking back now, who do you think plays an **important role** in preventing pregnancy related complications?  *Prompt: How do you think responsibilities distribute between patients, doctors and your significant other?* | **Social/Professional role and identity** |
| 30. Where there are any particular steps you needed to make in **order to keep up** with taking the medication?  What do you think will be helpful? | **Behavioural regulation** |

**Could you please summarise for me what do think were the main barriers for you to take aspirin as prescribed? And what made this easier?**

**Concluding Points**

- Tell the participant that you have covered all the questions outlined in the interview plan and ask participant whether they have anything else to add.
- Offer an opportunity to ask questions.
- Confirm that the participant is still content that their interview data contribute to the analysis.
- Ask the participant if they would like to review a copy of the interview transcript.
- Establish whether the participant would like to receive a copy of the study findings (explain that this may take some time).
- Confirm whether the participant requires a referral to birth reflections or Obstetric clinic following the interview.
- Thank the participant for their time and participation in the interview and wish the participant well.
